# Problematic online behaviors and their early patterns of co-occurrence in young adults: insights from directed and undirected networks

**DOI:** 10.3389/fpsyt.2025.1446338

**Published:** 2025-02-24

**Authors:** Marta Błoch, Błażej Misiak

**Affiliations:** Department of Psychiatry, Wroclaw Medical University, Wroclaw, Poland

**Keywords:** internet addiction, mental disorder, comorbidity, early intervention, network analysis

## Abstract

**Introduction:**

The study aimed to identify early patterns of clustering within problematic online behaviors (POBs), their dynamics, and associations with several domains of psychopathology in young adults without a history of psychiatric treatment.

**Methods:**

Participants (n = 1441, aged 29.5 ± 6.3 years, 51.4% females) completed self-report measures recording the level of various POBs and several domains of psychopathology. Various approaches were used to analyze the data, including a principal component analysis together with the analysis of partial correlation networks (undirected associations) and Bayesian networks (directed associations).

**Results:**

Three distinct communities of variables were identified, including two communities of POBs (the first one: problematic use of social networking, problematic online shopping, and cyberchondria; the second one: problematic online gaming and gambling, cybersex) and one community of psychopathology. However, correlations between specific POBs were small to moderate. Problematic use of social networking sites, obsessive-compulsive disorder (OCD) symptoms, and problematic online gaming were found to be the bridge nodes. However, OCD symptoms were most likely to predict other POBs (all POBs except for cybersex). In turn, problematic use of social networking sites predicted the greatest number of other POBs (cyberchondria, gaming, and gambling).

**Discussion:**

These findings suggest that POBs tend to cluster into specific comorbidity patterns while remaining distinct entities. The symptoms of OCD are most likely to trigger the occurrence of POBs. Among POBs, problematic use of social networking sites might be most likely to predict the emergence of other POBs.

## 1 Introduction

Due to continuous development of the cyberspace and the increasing number of internet users, new phenomena related to excessive use of the internet have become more frequent in recent years. The most active users of the internet are adolescents and young adults, likely due to the fact that they grew up in an era where well-developed internet was readily accessible (1). This demographic group, comprising those born after 1984, also known as the *digital generation* or *digital natives* (2), has been found to be at a higher risk of developing internet use disorders. In an increasing number of countries, the problem has reached the magnitude of a significant public health concern, as more and more new negative health and psychosocial consequences are observed and documented.

Recent studies support the view that internet addiction or problematic internet use are not a single phenomenon but a spectrum of internet-related disorders (3). Indeed, problematic online behaviors (POBs) include problematic online gaming, cyberchondria, problematic cybersex (i.e., problematic online pornography use), problematic online shopping, problematic use of social networking sites (i.e., problematic social media use), problematic online gambling, cyberbullying, and digital hoarding (4). However, the first six of these POBs are best documented in terms of negative consequences and associated functional impairment (5–10).

The relationships between POBs, their co-occurrence and features predisposing internet users to a specific disorder are still being explored. Baggio et al. (11), who used a network analysis approach, found that POBs are organized as a spectrum of related but distinct entities. However, it has still not been investigated which specific POBs tend to co-occur and what are the common mechanisms involved. Experts from the European network for problematic usage of the internet proposed a division of POBs into two groups: the first one related to impulsive behaviors (i.e., gaming, gambling, online shopping, cybersex, and social media use) and the other, probably more strongly related to compulsive behaviors (e.g., cyberchondria), although the occurrence of addictive, impulsive, and compulsive traits is acknowledged in all types of POBs (4).

Only two categories of POBs are posited as diagnostic entities in the 11th Edition of the International Classification of Disease (ICD-11), i.e., gaming disorder (6C51.0) and gambling disorder (6C50.1), both of them with “predominantly online” subtype. However, a novel diagnostic category - compulsive sexual behavior disorder (6C72), included in the impulse control disorders, may be used to diagnose problematic cybersex (4). Also, a diagnostic entity of other specified or unspecified disorders due to addictive behaviors has been developed and may cover some POBs, e.g., problematic online shopping or problematic use of social media. However, specific criteria for these POBs have not been developed so far.

It remains largely unknown what are the mechanisms underlying the comorbidity of POBs and other mental disorders. Previous research has demonstrated that attention-deficit/hyperactivity disorder (ADHD), obsessive-compulsive disorder (OCD), anxiety disorders and depression, social phobia, impulse dysregulation as well as dissociative symptoms are risk factors for behavioral addictions, including broadly defined internet addiction (12). However, since it is known that assessment of POBs should not lump them together under the umbrella term of *internet addiction*, researchers focus more on predictors of specific POBs. For instance, it has been found that problematic online gaming is associated with increased levels of depression, anxiety, ADHD symptoms, and OCD symptoms (13, 14). In turn, higher levels of health anxiety, OCD symptoms and intolerance of uncertainty have been associated with cyberchondria, while higher levels of depressive symptoms might predict cyberchondria severity (15). Problematic cybersex, online shopping, and gambling have been associated with high levels of anxiety and depression (7, 13, 16, 17). Problematic use of social networking sites has often been associated with symptoms of anxiety, depression and ADHD (9). Finally, Starcevic et al. conducted a study in a sample of the Australian general population in which they examined frequency rates and predictors of POBs (18). They found that the level of ADHD symptoms predicts the occurrence of all POBs. Anxiety and depression levels appeared to be correlated with POBs, but they were not independent predictors of POBs in the context of other key variables. The study was the first to show a significant association between ADHD symptoms and a broad spectrum of POBs.

It should be noted that previous studies have mainly investigated the associations between single POBs and psychopathological domains using predefined models of causality. The area of studies investigating a wide range of POBs and their early patterns of co-occurrence has not been fully understood or systematically explored. The present study is exploratory in nature, which is particularly appropriate in such contexts, as its priority is to generate evidence, identify patterns, and formulate potential questions or hypotheses for future research, rather than to test specific, pre-established hypotheses about causal relationships. The development of analytical approaches to study such patterns has provided a new tool, known as a network analysis (19–22). It allows to investigate a single model of variables (nodes) without imposing specific directions of tested associations. Moreover, a network analysis provides opportunity to indicate variables that are critical (in other words the most central; using terminology that is specific for a network theory) for the occurrence of other phenomena tested in the network. This opens up a unique opportunity to indicate the most effective targets for interventions. A growing number of studies on internet-related disorders have used this type of analysis, but few have focused on specific POBs (11, 23–26). In light of these considerations, aims of the present study were threefold. First, we explored whether specific POBs cluster together in order to inform about potential comorbidity patterns. Second, using a network analysis, we aimed to test the associations between specific POBs and various domains of psychopathology. Third, we explored which variables are the most central in the network and thus are most likely to activate the associations between POBs and psychopathology. In order, to recognize early patterns of co-occurrence, we focused our study on individuals without a prior history of psychiatric treatment.

## 2 Methods

### 2.1 Participants

In March 2024, a study was conducted within a community sample from Poland by the computer-assisted web interview. Participants were surveyed through an online platform used for research surveys. Criteria for inclusion were being aged between 18 and 40 and having no history of psychiatric treatment, which was assessed through the following question: “Have you ever received any psychiatric treatment?”. The recruitment methods were designed to ensure that the demographic characteristics of participants are closely matched to those of the general Polish population, particularly in terms of age and gender. Eligible individuals received a survey link containing self-report questionnaires. Before completing the survey, participants were informed about its confidentiality and anonymous character of the data collection process. Responses that were incomplete or significantly deviated from the norm were filtered out by the platform. All participants agreed to participate in the survey. The study protocol received approval from the Bioethics Committee (approval number: 240/2024).

### 2.2 Measures

Participants were asked to complete a demographic questionnaire and self-report questionnaires. Demographic information included age, gender, education level, employment status, place of residence, relationship status, and a monthly income. Self-report questionnaires recorded the presence of POBs and psychopathological symptoms. Among POBs, the following behaviors were assessed: problematic use of social networking sites, problematic online shopping, problematic online gaming, problematic online gambling, cybersex, and cyberchondria.

To measure problematic online gaming, we used a validated Polish version of the Internet Gaming Disorder Scale–Short-Form (27). In turn, a problematic use of social networking sites was assessed using the Internet Addition Test which has been translated into Polish and has been shown to have good psychometric properties (28). This questionnaire has been adapted for each of the POBs (29–31). In the case of cybersex and online shopping, we used a short version of the Internet Addiction Test (32). Polish version of the Problem Gambling Severity Index is an accepted and widely used tool to screen for problematic online gambling. The Cyberchondria Severity Scale Short-Form was used to assess the severity of cyberchondria (33). Its Polish version was translated by Bajcar et al. (34), and shows good psychometric properties.

The measures of psychopathology covered the symptoms of ADHD, depression, mania, anxiety and OCD as well as psychotic-like experiences (PLEs). We have used tools that have been validated and are known to be reliable. Detailed information about specific measures used in the present study is provided in **Table 1**.

**Table 1:** The measures of problematic online behaviors and psychopathology used in the present study.

Category of measures	Variable	Questionnaire	Description of the questionnaire	Cut-off score	Cronbach’s alpha
Problematic online behaviors	Problematic online gaming	IGDS9-SF (27)	9 items; 5-point scale (1 – ‘never’; 5 – ‘very often’); total score: 9 – 45	> 32	0.92
	Problematic cybersex	SIAT-SE (28, 29)	12 items; 5-point scale (1 – ‘never’; 5 – ‘very often’); total score: 12 - 60	> 41	0.93
	Problematic online shopping	SIAT-SH (28, 30)	12 items; 5-point scale (1 – ‘never’; 5 – ‘very often’); total score:12 - 60	> 38	0.93
	Problematic use of social networking sites	IAT-SNS (28, 31)	18 items; 5-point scale (1 – ‘never’; 5 – ‘very often’); total score: 18 - 90	> 56	0.96
	Problematic online gambling	PGSI (62)	9 items; 4-point scale (0 – ‘never’; 3 – ‘always’); total score: 0 - 27	> 7	0.96
	Cyberchondria	CSS-12 (33, 34)	12 items; 5-point scale (1 – ‘never’; 5 – ‘very often’); total score: 12 - 60	> 49	0.90
Psychopathology	Psychotic-like experiences (PLEs)	PQ-16 (63)	14 items^*^; 2-point scale (‘true’/’false’); total score: 0 - 14	–	0.78
	Depressive symptoms	PHQ-9 (64)	9 items; 4-point scale (0 - ‘never’; 3 -’nearly every day’); total score: 0 - 27	–	0.87
	Manic symptoms	MDQ (65, 66)	13 items; 2-point scale (‘yes’/’no’); total score: 0 - 13	–	0.85
	Obsessive-Compulsive Disorder symptoms	OCI-R (67, 68)	18 items; 5-point scale (1 – ‘never’; 5 – ‘very often’); total score: 0 - 72	–	0.92
	Anxiety symptoms	GAD-7 (69)	7 items; 4-point scale (0 - ‘never’; 3 - ‘nearly every day’); total score: 0 - 21	–	0.93
	Attention-Deficit/ Hyperactivity Disorder symptoms	ASRS-5 (70)	6 items; 5-point scale (0 - ‘never’; 4 - ‘very often’); total score: 0 - 24	–	0.78

ASRS-5, the Adult ADHD Self-Report Scale for DSM-5; CSS-12, the Cyberchondria Severity Scale Short-Form; GAD-7, the Generalized Anxiety Disorder-7; IAT-SNS, the Internet Addition Test modified for use of social networking sites; IGDS9-SF, The Internet Gaming Disorder Scale–Short-Form; MDQ, the Mood Disorder Questionnaire; OCI-R, the Obsessional Compulsive Inventory – Revised; PGSI, the Problem Gambling Severity Index; PHQ-9, the Patient Health Questionnaire-9; PQ-16, the Prodromal Questionnaire-16; SIAT-SE, The Short Internet Addiction Test – Sex; SIAT-SH, the Short Internet Addiction Test – Shopping.

^*^To avoid potential overlap the PHQ-9 and GAD-7 scales, two items (1 and 7) were excluded as they might measure depressive and anxiety symptoms.

### 2.3 Data analysis

Only complete survey questionnaires were analyzed (i.e., there were no missing data). In the first step, we performed a principal component analysis of various POBs and domains of psychopathology in order to assess whether they cluster together and form separate communities. The number of dissected components was based on the analysis of a scree plot and eigenvalues > 1. The oblique promax method was used for factor rotation. This part of data analysis was carried out using the JASP 0.17 software.

In the second step, the undirected network of partial correlations was analyzed in the R software (version 4.1.3) using the following packages: *networktools* (35), *bootnet* (36), *qgraph* (37), and *mgm* (38). The network was limited to continuous variables and thus it was estimated using the EBICglasso approach (36). It is based on the use of the Least Absolute Shrinkage and Selection Operator (LASSO) that allows to regularize the network and avoid indicating weak associations (39). The network shows specific variables visualized as nodes that are connected with edges. Thicker edges indicate stronger partial correlations. Green edges depict positive correlations while red edges illustrate negative correlations. Next, the node centrality was assessed by calculating the bridge expected influence (40). It shows the sum of positive and negative edges between a specific node and all nodes from other communities. The communities were defined according to results of the principal component analysis. A higher bridge expected influence indicates a greater importance of a specific node. Finally, we analyzed the network accuracy, stability and the significance of between-edge differences in their weights (36). This part of data analysis was based on case-drop and non-parametric bootstrapping with 1,000 iterations. Results of the network analysis were considered stable if the correlation stability coefficient was higher than 0.25 (ideally it should be higher than 0.50) (36).

Importantly, the most central nodes in the partial correlation networks can be perceived as the most therapeutic targets only if they predict the occurrence of other variables in the network (41). As this point cannot be addressed using partial correlation networks, in third step of data analysis, the Bayesian networks based on directed acyclic graphs (DAGs) were analyzed (29). Indeed, DAGs inform about directional probabilities between nodes. The Incremental Association Markov Blanket (IAMB) was used to compute DAGs (42). More frequently appearing edges show a greater strength and directional probability. A non-parametric bootstrapping based on 2000 iterations was used to ensure stability of the network. The resulting network was averaged to show the associations observed in more than 50% of models. This part of data analysis was performed using the *bnlearn* R package (43).

## 3 Results

### 3.1 General characteristics of the sample

A total of 2775 individuals were approached for participation. Among them, 659 individuals (23.8%) reported a lifetime history of psychiatric treatment and were excluded. In turn, 635 individuals (22.9%) declined to participate in the survey, and 40 individuals did not complete the whole survey (1.4%). Finally, 1441 individuals (51.9%) participated in the survey (aged 29.5 ± 6.3 years, 51.4% females). The general characteristics of this sample are shown in **Table 2**. Participants were most likely to report a higher education level (43.3%), full-time employment (52.9%), urban place of residence (63.7%), single marital status (41.1%), and the monthly income equivalent to 750 – 1,250 USD (39.2%). Among specific POBs, participants were most likely to report a positive screening for problematic use of social networking sites (12.1%). Least frequently, they reported a positive screening for cyberchondria (0.8%).

**Table 2:** Descriptive characteristics of the sample (n = 1441).

	Mean ± SD or *n* (%)
Age, years	29.5 ± 6.3
Gender	
*Male*	701 (48.6)
*Female*	740 (51.4)
Education	
*Primary*	91 (6.3)
*Vocational*	109 (7.6)
*Secondary*	617 (42.8)
*Higher*	624 (43.3)
Employment	
*Unemployed*	168 (11.7)
*Part-time work*	193 (13.4)
*Full-time work*	763 (52.9)
*Student*	312 (21.6)
*Other*	5 (0.3)
Place of residence	
*Rural*	523 (36.3)
*Urban (up to 50,000 inhabitants)*	331 (23.0)
*Urban (50,000–150,000 inhabitants)*	161 (11.2)
*Urban (150,000–500,000 inhabitants)*	178 (12.4)
*Urban (>500,000 inhabitants)*	248 (17.2)
Income	
*< 750 USD*	331 (23.0)
*750 – 1,250 USD*	565 (39.2)
*1,250 – 1,750 USD*	211 (14.6)
*1,750 – 2,500 USD*	69 (4.8)
*> 2,500 USD*	33 (2.3)
*Refusal to answer*	232 (16.1)
Maritial status	
*Married*	475 (33.0)
*Informal relationship*	349 (24.2)
*Single*	275 (41.1)
*Divorced*	25 (1.7)
IGDS9-SF, score	16.5 ± 6.4
IGDS9-SF, positive screening	30 (2.1)
SIAT-SE, score	19.4 ± 8.1
SIAT-SE, positive screening	16 (1.1)
SIAT-SH, score	20.2 ± 8.1
SIAT-SH, positive screening	61 (2.6)
IAT-SNS, score	37.7 ± 14.5
IAT-SNS, positive screening	174 (12.1)
PGSI, score	5.5 ± 5.4
PGSI, positive screening	86 (6.0)
CSS-12, score	27.7 ± 8.7
CSS-12, positive screening	12 (0.8)

IGDS9-SF, The Internet Gaming Disorder Scale–Short-Form; SIAT-SE, The Short Internet Addiction Test – Sex; SIAT-SH, the Short Internet Addiction Test – Shopping; IAT-SNS, the Internet Addition Test modified for use of social networking sites; PGSI, the Problem Gambling Severity Index; CSS-12, the Cyberchondria Severity Scale Short-Form.

### 3.2 The principal component analysis

**Figure 1:**
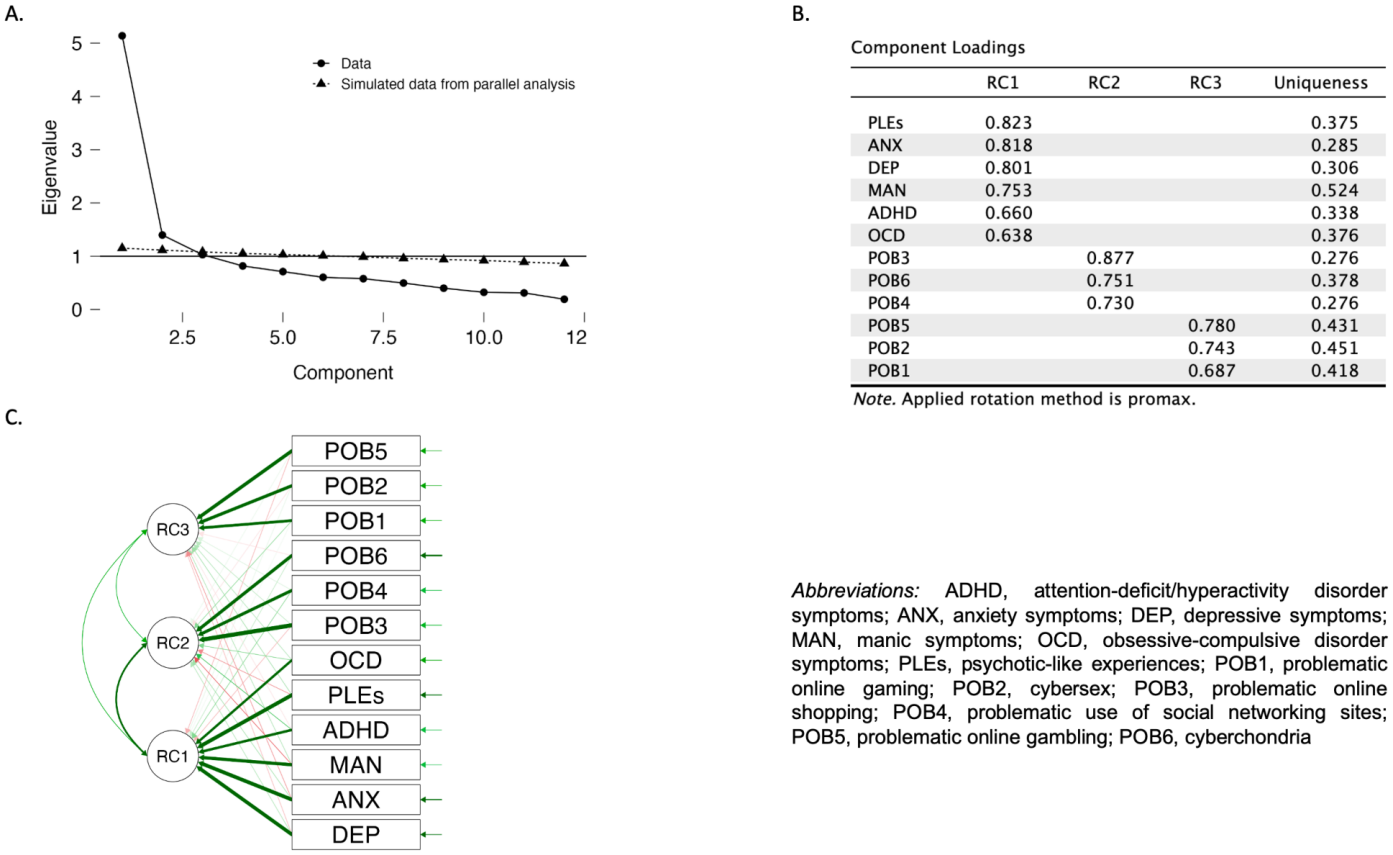
Results of the principal component analysis: **(A)** the scree plot; **(B)** component loadings; **(C)** the path diagram.

Results of the principal component analysis are shown in **Figure 1**. Altogether, three distinct communities were identified, including two communities of problematic online behaviors and one community of psychopathology. The first community of problematic online behaviors covered problematic online gaming, cybersex, and problematic online gambling. In turn, the second one included problematic online shopping, problematic use of social networking sites, and cyberchondria.

### 3.3 The network of partial correlations (undirected)

**Figure 2:**
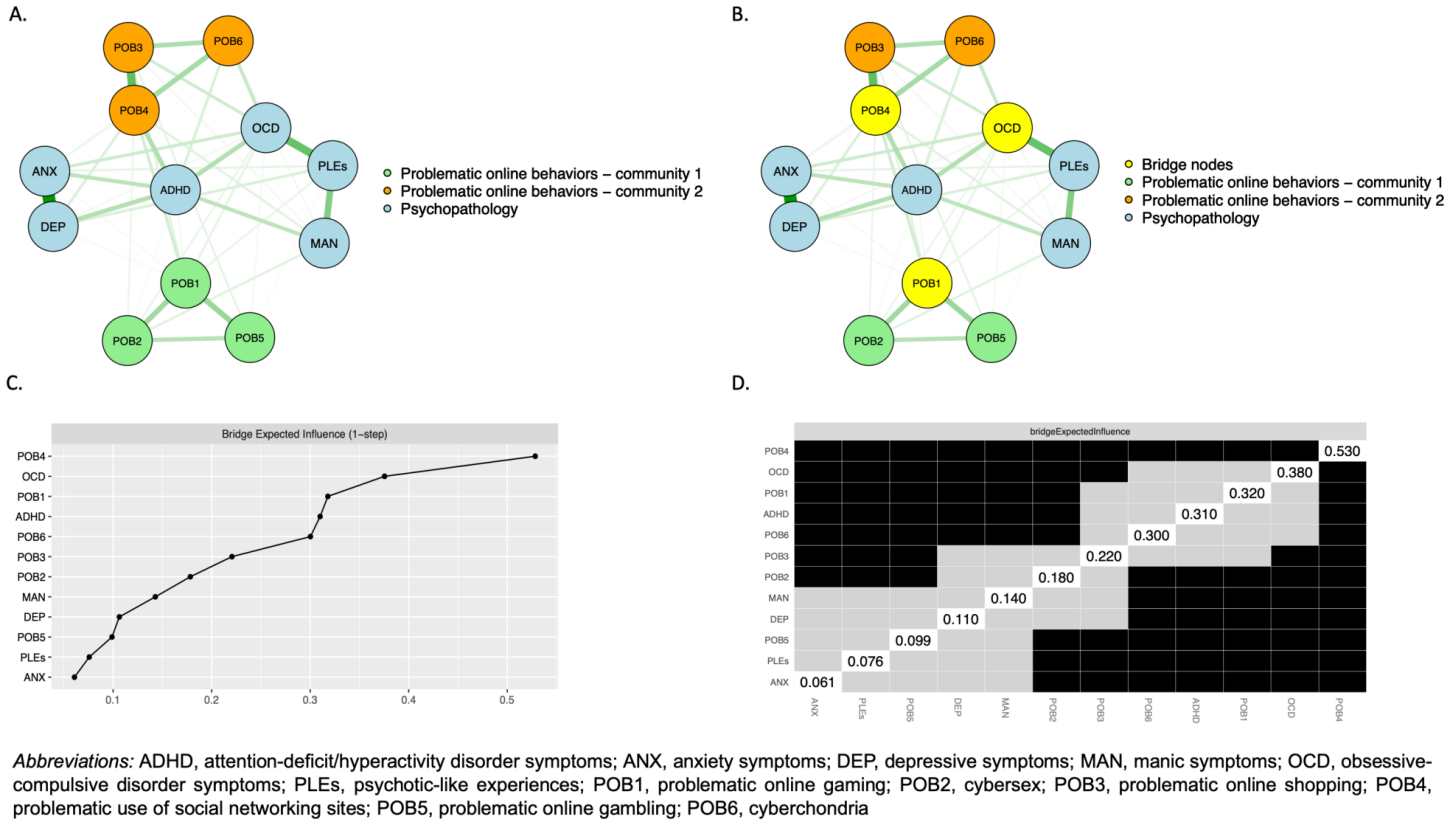
The network analyzed in the present study (thicker edges indicate stronger associations; green edges show positive associations while red edges show negative ones) and corresponding bridge expected influence metrics: **(A)** the network of problematic online behaviors and domains of psychopathology; **(B)** the network of problematic online behaviors and domains of psychopathology with nodes showing the highest bridge expected influence (yellow nodes); **(C)** the bridge expected influence metrics; **(D)** the comparison of bridge expected influence metrics (black boxes show significant between-node differences).

The network analyzed in the present study is shown in **Figure 2A**. Altogether, 41 edges had non-zero weights (out of 66 potential edges, 65.2%). All nodes appeared to be well-connected and only two edges had negative weights (**Table 3**). Three nodes were found to be the bridge nodes, i.e., their bridge expected influence was ranked among top 20% bridge expected influence values in the whole network (**Figures 2B, C**). These nodes included those representing problematic use of social networking sites, OCD symptoms, and problematic online gaming. However, the bridge expected influence of problematic use of social networking sites was significantly higher compared to the bridge expected influence metrics of all other nodes in the network (**Figure 2D**). Higher levels of problematic use of social networking sites were significantly associated with higher scores of depressive and anxiety symptoms, manic symptoms, and ADHD symptoms. In turn, higher levels of problematic online gaming were significantly associated with higher scores of depressive symptoms, ADHD symptoms, PLEs, and OCD symptoms as well as lower scores of anxiety symptoms. Finally, OCD symptoms were significantly correlated with scores of all problematic online behaviors, except for problematic use of social networking sites.

**Table 3:** Weights matrix.

	POB1	POB2	POB5	POB3	POB4	POB6	DEP	ANX	MAN	ADHD	PLEs
POB1	–										
POB2	0.222	–									
POB5	0.237	0.172	–								
POB3	0.067	0.000	0.057	–							
POB4	0.106	0.044	0.000	0.376	–						
POB6	0.000	0.013	0.000	0.191	0.212	–					
DEP	0.021	0.000	0.000	0.000	0.085	0.000	–				
ANX	–0.017	0.000	0.000	0.000	0.038	0.039	0.600	–			
MAN	0.000	0.072	0.000	–0.026	0.069	0.028	0.000	0.000	–		
ADHD	0.031	0.003	0.000	0.000	0.186	0.090	0.180	0.157	0.155	–	
PLEs	0.051	0.000	0.013	0.012	0.000	0.000	0.090	0.059	0.288	0.000	–
OCD	0.059	0.046	0.029	0.111	0.000	0.131	0.031	0.116	0.000	0.183	0.359

ADHD, attention-deficit/hyperactivity disorder symptoms; ANX, anxiety symptoms; DEP, depressive symptoms; MAN, manic symptoms; OCD, obsessive-compulsive disorder symptoms; PLEs, psychotic-like experiences; POB1, problematic online gaming; POB2, cybersex; POB3, problematic online shopping; POB4, problematic use of social networking sites; POB5, problematic online gambling; POB6, cyberchondria.

Three connections between psychopathology and problematic online behaviors with the highest edge weights were as follows: (1) between ADHD symptoms and problematic use of social networking sites; (2) between OCD symptoms and cyberchondria, and (3) between OCD symptoms and problematic online shopping (**Table 3**). The first two of these connections did not differ significantly with respect to corresponding weights (**Supplementary Figure 1**). However, the connection between ADHD symptoms and problematic use of social networking sites was significantly stronger compared to the connection between OCD symptoms and problematic online shopping as well as other connections between psychopathology and problematic online behaviors. However, it should also be noted that all weights for edges between psychopathology and problematic online behaviors were lower than 0.300 indicating (very) small strength of observed associations.

The CS-C value for edges and the bridge expected influence centrality was 0.75 (the same value for both network characteristics) suggesting sufficient network stability. Results of the bootstrapping procedures are shown in **Supplementary Figures 2–4**. Bootstrapped edge weights (**Supplementary Figure 3**) and bridge expected influence metrics (**Supplementary Figure 4**) largely overlapped with those observed before non-parametric bootstrapping.

### 3.4 The Bayesian network (directed)

**Figure 3:**
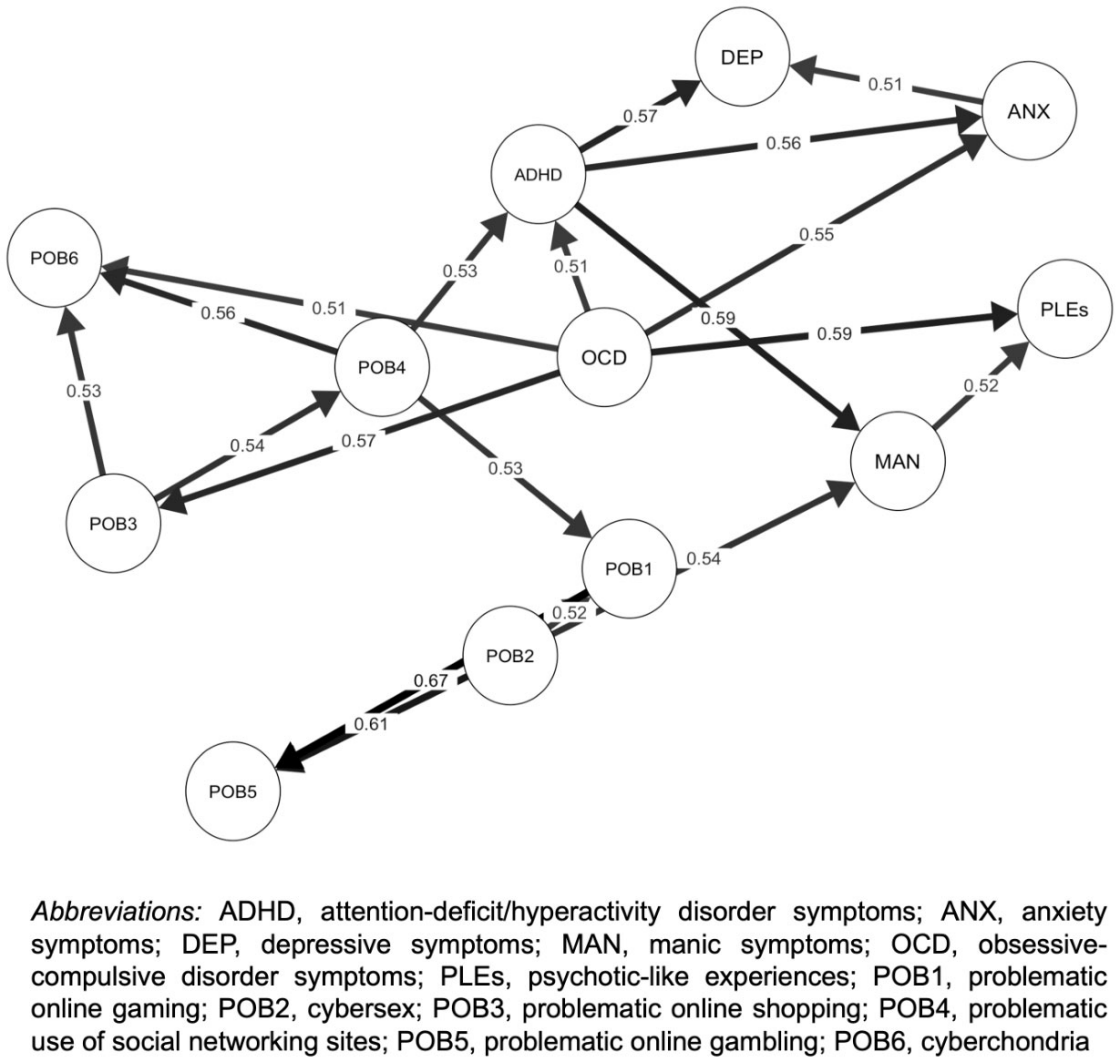
Directed acyclic graphs (DAGs) showing associations between psychopathological symptoms and problematic online behaviors. Arrows indicate the most likely directions of effects. Corresponding values refer to the percentage of models in which specific directed associations were observed. The figure shows only those associations that were present in more than 50% of models.

In this part of data analysis, our aim was to provide insights into the probability of directional associations of the bridge nodes with other nodes. A total of 19 directed arcs (out of 144 potential connections, 13.2%) were observed at the threshold of direction probability > 50%. This part of data analysis revealed that OCD symptoms are the only domain of psychopathology that might predict the emergence of POBs (**Figure 3**). In more than 50% of tested DAGs, OCD symptoms directly predicted the occurrence of problematic online shopping and cyberchondria. However, OCD symptoms were also likely to indirectly predict other POBs, including problematic use of social networking sites, gaming, and gambling, but not cybersex. Also, OCD symptoms were found to directly predict the levels of ADHD symptoms, anxiety, and psychotic-like experiences. In turn, problematic use of social networking sites was found most likely to predict (directly or indirectly) cyberchondria, problematic online gaming and gambling as well as ADHD symptoms. Finally, problematic online gaming was most likely to only predict problematic online gambling. The Bayesian network analysis also demonstrated that POBs tend to cluster together within two communities dissected using the principal component analysis.

## 4 Discussion

To date, relatively few studies have explored the broader spectrum of POBs and their co-occurrence (11, 44–46). However, these studies have examined diverse populations and employed varying psychometric instruments, which may contribute to inconsistent findings. Furthermore, these studies have primarily focused on demonstrating that the symptoms of individual POBs represent distinct constructs, thereby reinforcing the notion that the term ‘Problematic Internet Use’ is conceptually imprecise and potentially misleading. The main findings of the present study indicate that POBs might form two distinct clusters in young adults. The first one is composed of problematic online gaming, cybersex, and problematic online gambling. In turn, the second one might include problematic online shopping, problematic use of social networking sites, and cyberchondria. A comparable cluster was identified in the study conducted by Baggio et al. (11), who employed a methodological approach similar to the one used in the present study. In their network analysis, problematic use of social networking sites and problematic online shopping appeared to be directly interrelated. Similar findings were observed for problematic online gaming and gambling. These findings support the view that, although they primarily form separate constructs, POBs are likely to co-occur in specific comorbidity clusters. This finding provides implications for clinical practice indicating the necessity of awareness towards emerging comorbidity within POBs. Similar patterns have been previously observed with respect to gender differences, where men have been found to be more likely to become addicted to online video games, cyber pornography, and online gambling, while women have been observed to develop addictive use of social media and online shopping (47–53). Also, a recent network analysis addressing the spectrum hypothesis of POBs revealed that problematic online shopping, problematic social networking, and cyberchondria might be most closely interrelated (11). However, the authors also observed relatively small correlation coefficients between specific POBs, supporting the conceptualization that specific POBs might serve as separate constructs. This is also in line with our observations, where POBs appeared to be interrelated with small-to-moderate correlation coefficients. Indeed, the greatest correlation coefficient within specific POBs was observed between problematic online shopping and problematic social networking.

Another important observation that originates from our study is that problematic social networking, OCD symptoms, and problematic online gaming appeared to be the bridge nodes. However, a Bayesian network analysis revealed that OCD symptoms might predict almost all POBs (except for cybersex) which is in line with previous reports (8, 12–15, 54, 55) In turn, problematic use of social networking sites appeared to predict POBs to a lesser extent (direct and indirect probabilities of predicting cyberchondria, problematic online gaming and gambling) while problematic online gaming predicted problematic online gambling only. Importantly, behavioral addictions have been found highly prevalent among individuals with OCD. For instance, the analysis of data from 6916 treatment-seeking individuals with OCD estimated the prevalence of problematic internet use at 8.7% (56). It has also been found that substance use disorders, gaming disorders, and OCD might share overlapping neurofunctional alterations (57). Previous studies have also demonstrated that problematic social networking is a transdiagnostic phenomenon, i.e., it might occur in the context of various, unspecific domains of psychopathology including depression, anxiety, OCD, and ADHD (58). It is likely that these associations reflect shared neurofunctional impairments. Indeed, it has been reported that problematic use of social networking sites is associated with impaired inhibitory mechanisms and reduced grey matter across several brain regions including the amygdala, nucleus accumbens, and insula (59).

Although our study was not primarily designed to assess prevalence rates of POBs in the Polish population, these aspects also need to be discussed. Altogether, we observed that a positive screening for problematic use of social networking sites had the highest prevalence rate reaching 12.1%. The prevalence rates for other screened constructs of POBs were as follows: 6.0% for problematic online gambling, 2.6% for problematic online shopping, 2.1% for problematic online gaming, 1.1% for cybersex, and 0.8% for cyberchondria. Of note, these prevalence rates might be lower compared to other studies based on representative samples as we excluded individuals with a lifetime history of psychiatric treatment. For instance, the internet-based survey of POBs performed in 1626 individuals revealed the highest prevalence rate of problematic online shopping (i.e., it reached 12.2%) (18). Prevalence rates for other POBs ranged between 4.6% for cyberchondria and 11.4% for problematic online gambling. However, previous meta-analyses have revealed relatively similar prevalence rates as compared to those reported in our study. For instance, the prevalence of social media addiction based on severe level or strict polythetic classifications was found to be 13.0% (95CI: 8.0 – 19.0%) (60). Another meta-analysis estimated the prevalence of generalized internet addiction and internet gaming disorder at 7.02% (95%CI: 6.09% - 8.08%) and 2.47% (95%CI: 1.46% - 4.16%), respectively (61). However, the authors found that the prevalence of generalized internet addiction might increase over time. This effect appeared to be not significant for internet gaming disorder.

There are various limitations of the present study that need to be highlighted. First, the assessment of psychopathology and POBs was limited to self-reports. In this regard, findings may not be generalizable over clinical populations. Second, although the study covered a broad range of POBs, some of them were not assessed, e.g., short-form videos, cyberbullying, and digital hoarding. Third, insights into representativeness of our sample might be limited to some extent. Indeed, we excluded participants with a history of psychiatric treatment and did not record reasons of non-participation. However, our overarching aim was to recognize dynamics of POBs among individuals at risk of psychopathology and POBs. Moreover, our response rate reached 51.9%. Next, although Bayesian networks can provide probabilistic relationships and make predictions based on observed data, a cross-sectional design does not allow to provide insights into causal inferences. Finally, most of correlations were small-to-moderate strength and thus their clinical relevance should be interpreted with caution.

In sum, the present study indicates that POBs tend to co-occur in specific clusters. Moreover, we found that OCD symptoms are most strongly interrelated to various POBs and other co-occurring psychopathological symptoms. Importantly, OCD symptoms were observed to predict, either directly or indirectly, almost all POBs (except for cybersex). With respect to specific POBs, the present study revealed that problematic use of social networking sites is most likely to predict other POBs. Altogether, the study provides important implications for clinical practice by informing about most likely comorbidities among POBs and psychopathological symptoms. Moreover, the study informs about dynamics of POBs, where OCD symptoms and problematic use of social networking sites appear to be most likely to trigger the occurrence of other POBs. By focusing on these critical nodes, clinicians can leverage their centrality to achieve broader therapeutic outcomes, improving the overall efficiency, and effectiveness of treatment plan. Both early identification of OCD symptoms and psychoeducational programs aimed at informing about the healthy use of social media can reduce the likelihood of subsequent psychopathology associated with internet-related disorders, particularly in vulnerable populations, such as adolescents and young adults.

## Data Availability

The raw data supporting the conclusions of this article will be made available by the authors, without undue reservation.
