# Supplementary material for: Problematic online behaviors and their early patterns of co-occurrence in young adults: insights from directed and undirected networks
Source: Front Psychiatry. 2025 Feb 24;16:1446338. doi: 10.3389/fpsyt.2025.1446338 (PMC11891174; doi:10.3389/fpsyt.2025.1446338)
Supplement: Supplementary file 1 [file Supplementaryfile1.docx]

Supplementary Material

**Figure 1.** Bootstrapped differences between edge weights. Black boxes indicate significant differences between edge weights. *Note:* ADHD, attention-deficit/hyperactivity disorder symptoms; ANX, anxiety symptoms; DEP, depressive symptoms; MAN, manic symptoms; OCD, obsessive-compulsive disorder symptoms; PLEs, psychotic-like experiences; POB1, problematic online gaming; POB2, cybersex; POB3, problematic online shopping; POB4, problematic use of social networking sites; POB5, problematic online gambling; POB6, cyberchondria

**Figure 2.** Stability of the bridge expected influence centrality. The red line illustrates changes of the bridge expected influence centrality while dropping various proportions of data.


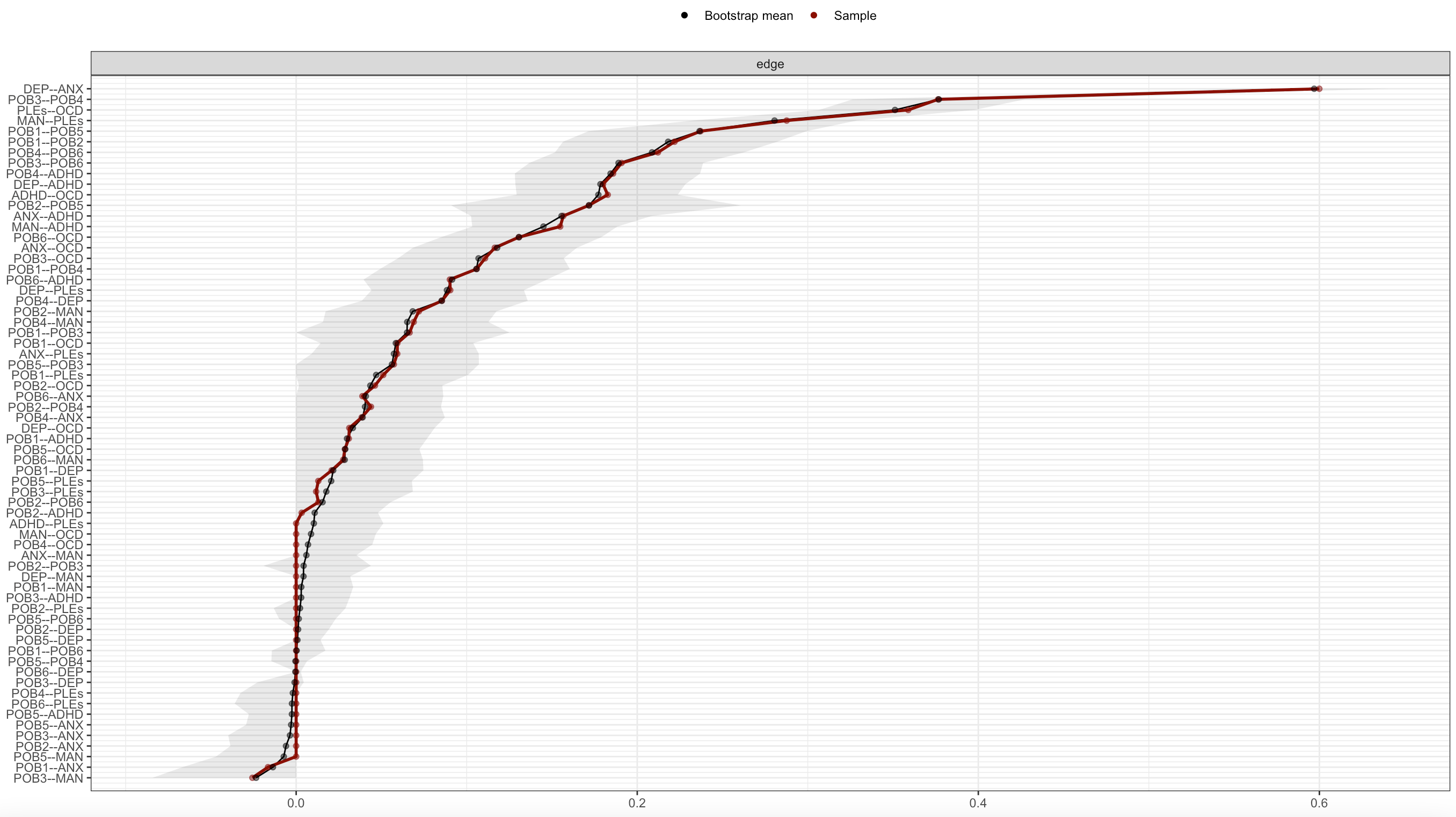


**Figure 3.** Bootstrapped 95% confidence intervals of edge weights. The sample values are illustrated by a red line. The bootstrapped 95% confidence intervals are shown within the grey area. *Note:* ADHD, attention-deficit/hyperactivity disorder symptoms; ANX, anxiety symptoms; DEP, depressive symptoms; MAN, manic symptoms; OCD, obsessive-compulsive disorder symptoms; PLEs, psychotic-like experiences; POB1, problematic online gaming; POB2, cybersex; POB3, problematic online shopping; POB4, problematic use of social networking sites; POB5, problematic online gambling; POB6, cyberchondria


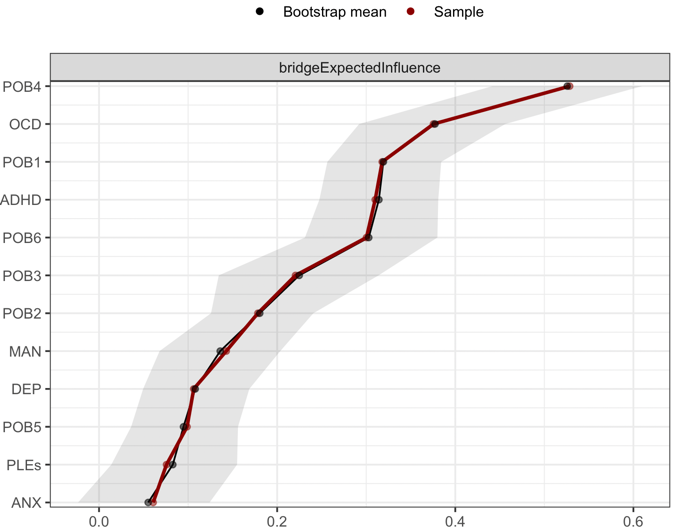


**Figure 3.** Bootstrapped 95% confidence intervals of bridge expected influence metrics. The sample values are illustrated by a red line. The bootstrapped 95% confidence intervals are shown within the grey area. *Note:* ADHD, attention-deficit/hyperactivity disorder symptoms; ANX, anxiety symptoms; DEP, depressive symptoms; MAN, manic symptoms; OCD, obsessive-compulsive disorder symptoms; PLEs, psychotic-like experiences; POB1, problematic online gaming; POB2, cybersex; POB3, problematic online shopping; POB4, problematic use of social networking sites; POB5, problematic online gambling; POB6, cyberchondria
